# Script or style? Analysis of the relationship between teaching scripts and supervision style

**DOI:** 10.1371/journal.pone.0338902

**Published:** 2026-01-08

**Authors:** Julia Sader, Nadia M. Bajwa, Robin Lüchinger, Thomas Fassier, Marie-Claude Audétat, Mathieu Nendaz, Noelle Junod Perron

**Affiliations:** 1 Unit of Development and Research in Medical Education, Faculty of Medicine, University of Geneva, Geneva, Switzerland; 2 Department of Community Health and Medicine, Faculty of Medicine, University of Geneva, Geneva, Switzerland; 3 Department of General Pediatrics at the Children’s Hospital, Geneva University Hospitals in Geneva, Geneva, Switzerland; 4 Division of Internal Medicine for the Aged, Geneva University Hospitals, Geneva, Switzerland; 5 Centre for Interprofessional Simulation, Geneva, Switzerland; 6 Faculty of Medicine, University Institute for Primary Care, University of Geneva, Geneva, Switzerland; 7 Department of Medicine, Service of General Internal Medicine, Geneva University Hospitals (HUG), Geneva, Switzerland; 8 Medical Directorate and Quality, Geneva University Hospitals, Geneva, Switzerland; Universiti Sains Malaysia, MALAYSIA

## Abstract

**Purpose:**

Clinical teachers use both clinical and teaching scripts when supervising students. The aims of the study was: 1) to explore how teaching scripts are operationalized during supervision of clinical reasoning, and 2) to evaluate whether teaching scripts and their activation vary according to clinical teachers’ level of teaching experience.

**Materials and methods:**

A purposeful sample of 20 clinical teachers from different disciplines and levels of teaching experiences were invited to conduct a videotaped clinical supervision with a simulated resident involving a patient presenting with subacute lower abdominal pain. The session was followed by a semi-structured and a stimulated recall interview. Both were transcribed and analysed using deductive and inductive approaches.

**Results:**

Teaching scripts were operationalized into four clear supervision styles along two axes: the richness of the pedagogical dimension of teaching scripts and the flexibility of teaching approaches. Participants’ working context, prior clinical experience, teaching training opportunities and reflective skills seemed to determine these teaching styles. However, they did not vary according to the level of teaching experience.

**Conclusion:**

The results of this study shed light on how faculty development programs can guide and enhance clinical teachers’ supervision skills based on the analysis of their existing teaching scripts and supervision styles.

## Introduction

Clinical supervision is essential in both pre- and postgraduate medical training in order to promote professional development and patient safety [1,2]. Effective clinical supervision requires a safe and supportive environment, a trustful supervisory relationship, regular supervision, and feedback, as well as clinical teachers trained in helping learners to solve their problems [1].

Clinical teachers use clinical and pedagogical reasoning processes when supervising students and junior doctors in their daily practice [3]. Both clinical and pedagogical reasoning processes rely on scripts to organize and transform knowledge in a multidimensional and ongoing action-oriented process [3]. Scripts refer to some form of organized knowledge which professionals’ access to diagnose problems, select activities, and connect related concepts in order to choose relevant and targeted strategies for both clinical and teaching issues [4].

Illness scripts are “specialized knowledge structures” that link clinically relevant, information about general disease categories, specific examples of diseases, and conditions that enable diseases to develop in people [5]. They allow physicians to draw from organized knowledge structures to guide their reasoning during clinical encounters from the diagnostic to the management stages [5]. Clinical teachers rely on illness scripts to ensure appropriate patient care. They also use teaching scripts when they supervise students and junior doctors.

Teaching scripts result from the interaction between the teacher’s knowledge about the subject matter, the teaching strategies/methods and knowledge of the students [6]. They help teachers select the content and identify the teaching methods according to the learners’ level of training and training needs during both formal and informal teaching interactions. In medical education, the content of teaching scripts that are at play during clinical teaching [3,4] are:

- Knowledge of medicine and patients – both medical scientific knowledge and illness scripts are essential for clinical practice and clinical teaching. Clinicians recall specific patients connected with certain illness stages and treatment courses. Their knowledge of specific patients allows them to keep an eye on student and resident interactions with the patient and provide appropriate guidance.
- Knowledge of contexts -clinical teachers operate in a wide range of settings. Context influences the sorts of patients seen, the diseases treated, and the therapeutic options offered, all of which influence learning opportunities. This knowledge is therefore used not only to guide learners but also to diagnose and treat patients.
- Knowledge of pedagogy and learners – clinical teachers learn and grasp pedagogical strategies, such as: questions, case discussions, observation and feedback, and specific clinical teaching approaches. They should also be aware of their students’ prior knowledge and beliefs, and understand their needs, motivations, and abilities

Teaching scripts can be activated in either structured teaching sessions or in the workplace setting. These teaching scripts are described as informal, tacit, and often idiosyncratic. The activation process is monitored by metacognition and requires reflection before, during, and after teaching actions [3].

Unlike illness scripts, only a few studies have explored teaching scripts in terms of content, applicability, and variability. Regarding teaching content and style, Irby observed six experienced clinical teachers teaching during ward rounds and noticed that they all used different clinical scripts and teaching styles [4]. Another study reported that in response to two clinical vignettes, clinical paediatricians identified the same student errors and offered broadly similar teaching points, regardless of the level of teaching experience [7]. Cantillon showed that GPs prioritised the content to be presented and taught differently, and differed according to their level of knowledge of the subject, as well as their knowledge and beliefs about students, the learning environment, and teaching [8]. Finally, a last study reported that collaborative development and sharing of teaching scripts for topics commonly encountered in the hospital setting contributed to teachers’ professional development in terms of gains in local expertise, changes in teaching strategies, and establishment of a collegial spirit [9].

The studies mentioned above focused on teaching scripts used by physicians of one speciality, took place in one type of teaching context (structured teaching sessions or hypothetical clinical supervision) or solely explored one or two dimensions of teaching scripts. Other studies analysed how clinical teachers’ supervision styles varied according to perceived roles, contextual priorities or entrustment decisions [10–14]. Little is known about how these different dimensions of teaching scripts articulate with each other and are operationalised during supervision of clinical reasoning by clinical teachers with different levels of teaching experience and professional backgrounds. Experienced clinical teachers may be more thoughtful than novice ones in selecting the most suitable teaching aids and methods based on the characteristics of the students or the context [15]. Similarly, given the influence of context in the development of scripts, clinical teachers with different professional backgrounds may trigger differently the different dimensions of their teaching scripts. The purpose of this study was 1) to explore how clinical teachers’ teaching scripts are operationalized during supervision of clinical reasoning 2) to evaluate whether teaching scripts and their activation vary according to clinical teachers’ level of teaching experience and professional background.

## Materials and methods

### Setting and participants

A qualitative study was conducted at the Geneva University Hospitals, Switzerland. Using a purposeful sampling approach, twenty clinical teachers from different disciplines (family medicine, hospital medicine, gynaecology/obstetrics and paediatrics) and different levels of teaching experience (1–2 years, 3–5 years, > 5 years) were recruited on a voluntary basis through an email invitation followed by a phone call in case of no response.

### Procedure

We developed a clinical case scenario adapted to the different disciplines involving- a 16-year-old female patient presenting with subacute lower abdominal pain (Box 1) and trained three 6^th^ year medical students during a 2-hour session to play the role of a 1^st^ year resident presenting the case and answering the clinical teacher’s questions in a standardized way. We involved 6^th^ year medical students since they were more available than 1^st^ year residents to participate to the study and we knew by experience that they could easily portrayed the knowledge, skills and attitudes of a 1^st^ year resident. The number of interactions they had varied between five and nine (student 1 = 5, student 2 = 6 and student 3 = 9) and involved clinical teachers from various disciplines. The quality assurance was in place to account for any differences in the way the three students played the role in the simulation.

**Box 1. Scenario of the simulated clinical supervision.**

I saw a patient, she’s 16, no 17 she has a stomach-ache and she’s really not feeling well, she took paracetamol but it didn’t make much difference. It started a few days ago, but actually she’s had pain like this before, several times but it went away without her doing anything, that was 3 months ago. Ella has missed school a few times recently and her mother has left a note because she didn’t go to school yesterday and the day before she would like a medical certificate to excuse her absence. There’s a bit of pressure.

So the patient tells me that the pain is somewhere between cramps and heaviness, it’s more localized down there (shows the lower abdomen), but it can go up around the navel. She’s not very hungry and in fact she told me she avoids eating for fear of being in pain. She has no fever but may have been shivering last night (temperature not measured). She has no nausea. Concerning the pain, it started a few days ago, I can’t find out more,

She doesn’t take any medication regularly and doesn’t have any allergies, except for hay fever. In any case, she has to take anti-histamines when she has an attack but at the moment it’s calm. She still says that the stools are hard, they have been like that for a long time, but she goes to the toilet less often, about every 3–4 days. Before it was maybe every 2 days. She has no urinary complaints now. She has had 1–2 urinary tract infections in the past. She also says she is bloated and has quite a bit of gas.

This is what I have in the history. Oh yes, and she’s tired, she has trouble waking up in the morning.

Now the physical exam, her pulse is 78/min, her blood pressure 105/60, her temperature 37.6.

In regard to ear-nose-throat, oropharynx is quiet, no cervical adenopathy.

Cardiac and pulmonary auscultation is normal.

In the abdomen, the belly is soft, tender around and below the umbilical area and a little in the right lower quadrant, on the left more than on the right, I did not find any defence, the abdominal noises are rather increased but of normal tone, the liver is at the costal margin, the spleen I did not palpate, the renal chambers are soft. She has pain in her flanks on both sides, but she also tells me that she has had lower back pain since her teenage years, I think from early scoliosis.

Otherwise, there is nothing cutaneous and I have not done the neurological exam.

What do we do?

In the scenario, the standardized residents were trained to generate few diagnostic hypotheses, to prioritize a diagnosis of constipation in context of stress related to family problems, and to suggest patient discharge with laxative without consider further investigation (S1 Appendix A). Standardized residents were instructed to express little motivation to explore further investigation if challenged by the clinical teacher (S1 Appendix A). The duration of the clinical supervision lasted between 9 and 12 minutes.

For each participant (clinical teacher), the case presentation made by the standardized residents and the supervision session were videotaped. A research assistant (RA) then conducted a two-step debriefing session that was also videotaped using a semi-structured interview guide (S2 Appendix B) that consisted of 1) an oral debriefing 2) a stimulated recall interview as the participant watched and analysed the videotaped supervision session, during which the RA explored the intentions and strategies used in terms of the content covered and the pedagogical strategies used, as well as their reasoning. The stimulated recall interview is used to help the person interviewed to verbalise and explain the different processes of his/her mental actions since cognitive processes cannot be observed and can only be inferred from observable elements [16]. The interview guide was pre-tested with three clinical educators and slightly adapted. Videotaped case presentation, supervision sessions, the oral debriefing, and the stimulated recall were transcribed verbatim. Only the oral debriefing and the stimulated recall were used for the study.

The study was granted a waiver from approval by the Ethical Committee of the canton of Geneva. Participants provided written informed consent and agreed to anonymous analysis of their data. Participants were free to withdraw from the study at any time.

### Analysis

Data were analysed using Braun and Clarke’s reflexive thematic analysis (RTA) [17]. Thematic analysis (TA) is a method used to report patterns within data [18]. In comparison, RTA is applied to the study of data that captures people’s experiences, perspectives, and perceptions. It is distinguishable from other forms of thematic analysis because coding—understood as the identification of patterns across the dataset—is treated as an active, reflexive process that is inseparably linked to the researcher conducting the analysis [19]. It follows a six-phase process: (1) familiarization with the data, (2) generating initial codes, (3) searching for candidate themes, (4) reviewing themes, (5) defining and naming themes, and (6) producing the analytic narrative.

Investigators included five experienced clinical teachers working in different medical disciplines and two research assistants trained in cognitive or organizational psychology. No researchers worked with the participants at the time of the study.

In the first stage (*familiarization with the data*), all researchers read the first two transcripts and independently identified key themes and passages. Randomly paired investigators read, summarized, and analysed four different participants’ transcripts. During five group sessions, the investigators engaged in collective reflection to mitigate individual bias – their perceptions and comments were discussed and documented until all transcribed sessions were reviewed. An initial list of codes was developed deductively, drawing on Irby’s five dimensions of teaching (knowledge of medicine/patients, context, teaching, and learners), which served as sensitizing concepts (*generating initial codes*) [4]. However, the coding process was subsequently enriched inductively through iterative engagement with participant narratives, group discussions, and reflexive dialogue among the research team. The mixed “deductive–inductive” approach is epistemologically compatible with RTA, as reflexive thematic analysis acknowledges the researcher’s active role in constructing themes and permits theoretical frameworks to inform—but not constrain—the analytic process [20]. This approach allowed themes to emerge also directly from the data, ensuring that participant’s accounts shaped the analysis beyond the pre-existing framework. As analysis progressed, distinct patterns of supervision styles began to emerge across the dataset, intersecting with the dimensions of teaching scripts described by Irby (*searching for candidate themes, reviewing themes*) [4]. To deepen this understanding, the research team undertook a second analytic cycle, revisiting all transcripts to explore how these teaching scripts were articulated and operationalised in terms of supervision strategies, attitudes, and styles (*defining and naming themes*). Through paired re-coding and subsequent group discussions, the team critically examined how the initial coding (based on Irby’s dimensions) related to the inductively emerging supervision styles, thereby shaping coherent and meaningful themes (supervision styles). Because most of the researchers were clinical educators, their professional roles may have possibly influenced data interpretation by emphasizing practical applicability of teaching scripts. However, the fact that researchers worked in teams mixing clinical educators and psychologists may have reduce such role dominance. In addition, a reflexive journal was maintained to document decisions and assumptions throughout the process [21]. Finally, in alignment with Braun and Clarke’s sixth phase, the researchers collaboratively constructed the analytic narrative, situating these supervision styles within broader conceptual and pedagogical contexts, and ensuring that the final themes captured both the richness of participant accounts and their interpretive significance.

NJP generated coding which was crosschecked by five researchers (MN, MCA, JS, NB, TF) and did not require any major change, coding consensus was considered to be strong and NJP coded the remaining verbatim. Transcripts were coded using ATLAS.ti 3.15 software for qualitative data analysis. All translations of participants’ utterances are the authors’.

## Results

Out of 47 clinical teachers (CT) approached, twenty clinical teachers participated – 13 did not answer to the invitation and 14 declined by lack of availability. The debriefing and stimulated recall sessions lasted approximately 1–2 hours.

Participants’ sociodemographic, clinical/teaching experience and professional background are displayed in Table 1 and Appendix C (S3 Appendix C). There were more clinical teachers from family and internal medicine than from gynaecology-obstetrics and paediatrics. Most clinical teachers were trained in Geneva, Switzerland.

**Table 1:** Clinical teachers’ sociodemographic and clinical/teaching experiences.

Female (n)	9
Male (n)	11
Age (mean)	39.35 (SD 7.50)
Specialization (n)	
Family medicine	7
Internal medicine	7
Gynaecology-Obstetrics	3
Paediatrics	3
Teaching experience as clinical teacher (years)	
1–2 years (low)	6
2–4 (medium)	7
> 4 (high)	7
Place of training (pre and postgraduate)	
Geneva	12
Geneva and Switzerland	2
Switzerland	1
Other	5

In the following paragraphs, we will describe how the several dimensions of teaching scripts, were articulated with each other and operationalized during supervision of clinical reasoning. We will first start with the pedagogy dimension since it largely influenced how teaching scripts were operationalized, followed by the learner, context, medicine and patient dimensions.

### Pedagogy dimension

**Fig 1:**
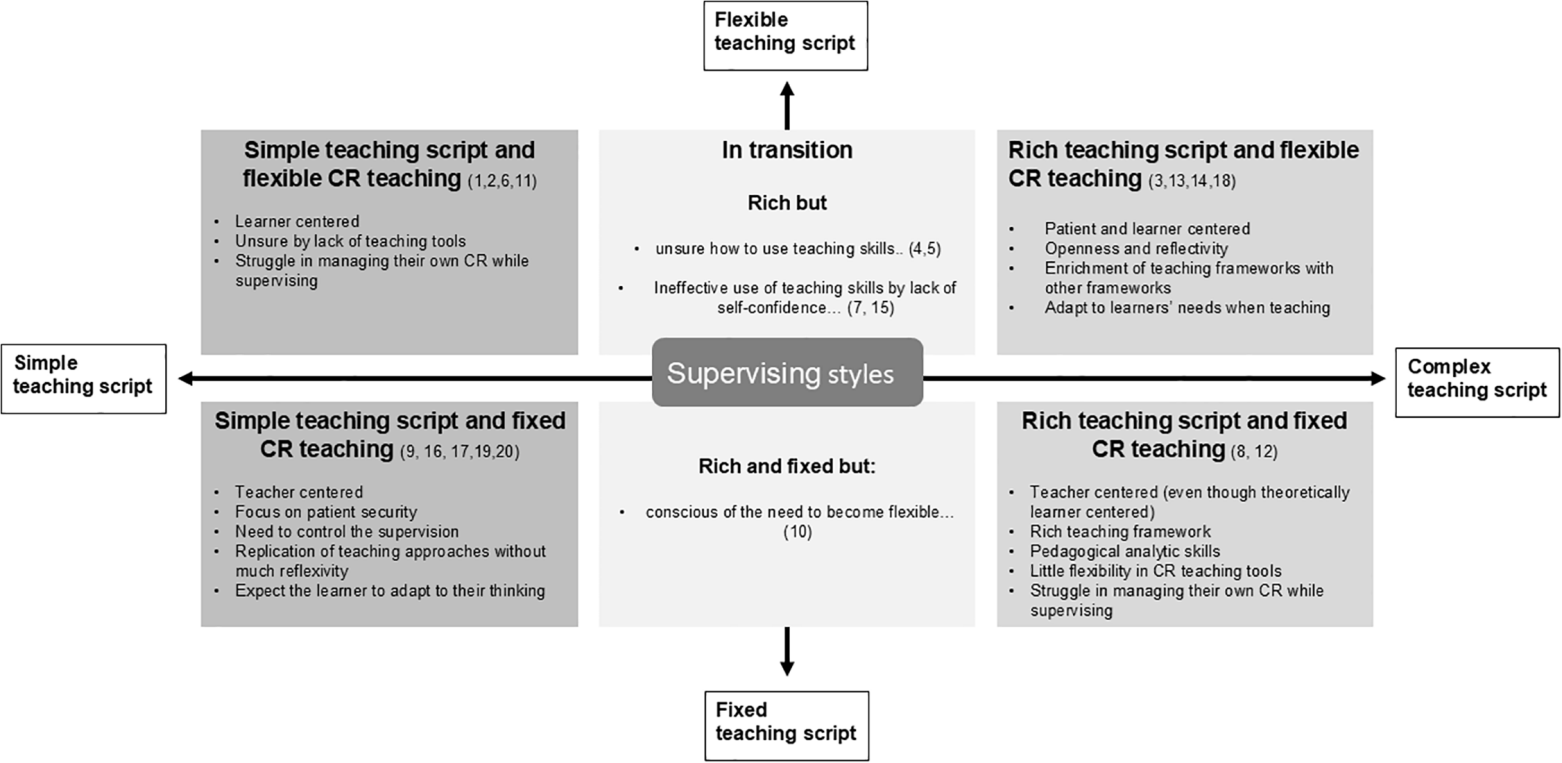
Operationalizing of the teaching scripts during supervision of clinical reasoning (numbers: clinical teachers’ (CT) identification).

Our analysis revealed that participants used different ways of organizing and applying their pedagogical knowledge and skills. To avoid thematic fragmentation, overlapping codes were reviewed collaboratively and grouped under umbrella themes [20]. We could classify such themes along two axes: the richness of scripts (simple vs rich), the supervising approach (fixed vs flexible). This allowed us to unveil four combinations of supervision styles that differed in terms of perceptions/ attitudes, understanding and behaviors regarding teaching intentions, learning culture, and clinical reasoning teaching (Fig 1). And some supervision styles that seemed to be in transition. Such differences were especially clear when participants expressed the various reasons why they would see the patient after the case presentation (Table 2). The supervision styles did not seem to be related to the level of teaching experience, but some were more represented in some disciplines than in others (S3 Appendix C).

**Table 2:** Specific reasons for seeing the patient according to participants’ supervision style.

Supervision style	Rationale for seeing the patient
Simple and flexible	To implicitly model both clinical and reasoning skills and work as a team with the learner. However, participants were not able to elaborate more on this issue.
Simple and fixed	To implicitly role model, to check for missing information, and to assess whether the clinical situation was urgent or not.
Rich and fixed	Not only to evaluate whether it was an emergency or not and to confront the learner but also to identify the learner’s problem and give feedback.
Rich and flexible	To give observation tasks to the learner, to demonstrate clinical skills, to debrief thereafter, and finally to reassure the patient.

#### Simple and flexible style.

In the simple and flexible style, participants (n = 4) were willing to be learner-centered, supportive but felt unsure on how to achieve this due to a lack of effective teaching tools as well as a clear teaching framework. However, they were aware of their difficulties and were reflective about them. They described replicating how they were taught or had wished to be taught:

*This is… one of my medical residents who taught me that skill when I was a junior during my residency. So, later on it stayed with me and I thought this method to be logical and effective. So, I learned that skill from him (CT12: line 40)*

They also struggled in managing their own clinical reasoning while supervising. Although they tried to build on learners’ clinical reasoning, they acknowledged that they needed to take control of the clinical reasoning process by synthesizing the clinical situation, questions, and challenges for themselves.

*“That’s what’s difficult, is that we try to create our own reasoning on the basis of what we’re told, so it requires an extra effort compared to if we see the patient directly. And then afterwards, things are brought to us that are a bit confusing and it’s difficult afterwards to stay on track because we feel a bit lost, we say to ourselves: “well, is this information relevant or not? And then... well, it has... well, it makes it difficult to reason clearer, I think.” (CT2: line 24)*

Going to see the patient afterwards was used to implicitly model both clinical and reasoning skills and work as a team with the learner. However, participants were not able to elaborate more on this issue.

#### Simple and fixed style.

Another group of participants (n = 5) shared a simple teaching framework, but their teaching approach seemed rather rigid and corrective. Their supervision intended to be structured, efficient, and focused essentially on solving the patient’s problem and ensuring patient security. Their use of teaching tools was largely implicit but reflected their need to control the supervision session; most questions were used to clarify, classify or verify:

*“In fact, I make a sort of list of what we need to catch up on - well, the next few days of the patient’s care. In fact, as I listen freely to this, I say to myself: “this is missing; this is missing; this is missing”, so that we can then complete and have well rounded structure. Meaning: a complete history and clinical examination, so that we can then say “it’s more like this diagnosis”. (CT9: line 33)*

Although they reported wanting to be tactful and to not upset the learner, some tended to use harsh words when talking to the learners about their difficulties and expressed negative emotions during the supervision. They tended to replicate without much reflexivity the teaching tools and approaches that they had experienced as learners themselves; several had been trained in a more authoritarian learning culture.

*“Then again, I’ve already seen residents who are missing… the medical skills, so we’re like: ‘Where did they even get their degree!?’ And well, some of them are hopeless, but some can be brought back. You just have to supervise them, push them a little, give them some books...”(CT20: line 69)*

In their vision of clinical reasoning teaching, the learner needed to adapt to their framework and structure of thinking. Clinical reasoning was described as key steps that they had to follow based on their own clinical reasoning framework they had developed through prior experience sometimes intuitively or through role modeling.

*“I tried to structure… I tried to go over it again… For the things that were missing in their history-taking, I tried to follow a sort of… how could I put it… a kind of framework for taking a history, really. That is, by focusing first on the pain, then on associated symptoms, then a kind of system-by-system history, the past medical history, etc. Trying to go over it using a kind of checklist.” (CT16: line 9)*

More gynecologists used a simple and fixed teaching style than other physicians.

#### Rich and flexible style.

A third group of participants (n = 2) was categorized as having a rich but fixed teaching style. Their intentions were clearly learner-centered and aimed at valuing the learners and developing their autonomy. They felt confident and expert in their field as both clinicians and teachers. They described a rich teaching framework nurtured by positive training experiences and explicit role models. These supervisors were able to accurately analyze the learner’s difficulties and to comment on the pros and cons of several teaching tools:

*“It was very tempting for me at one point not to use this technique at all and then to say: “well, he’s a mess, we’ll go through the systems again”. But I said to myself: “with someone who is just starting out, it’s likely to be even less useful, not only for him, but also for me because I’ll only get close ended answers.” (CT 8: line 47)*

However, their attitude during the supervision remained teacher-centered and they demonstrated little flexibility and variety in the use of teaching tools. Similarly, although their clinical reasoning was present and rich during the think-loud session, it was not explicitly displayed or shared during the supervision session. The focus of the clinical reasoning teaching was to be systematic and structured. They aimed at bringing the learner to their perspective and made little effort to adapt to the learner’s reasoning.

*“I can observe the fact that... that it’s going to be challenging, that it’s going to go in all directions because typically, (…) I know the reason for this consultation, the patient’s case and history (…), however, the patient is not following any logic which leaves me feeling uncomfortable and unable to follow the structure I have learned and have in mind.” (CT12: line16)*

Their reasons for seeing the patient expressed such duality. It was used not only to evaluate whether it was an emergency or not and to confront the learner but also to identify the learner’s problem and give feedback.

#### Rich and fixed style.

The last group (n = 4) was defined as having a rich and flexible teaching pattern. Their intention was to give room to the learner’s growth and development. They reported putting significant effort into making the learning atmosphere a trustful one, human-centered, and positive. Their attitude was open and reflexive. They described diverse learner-centered teaching tools in a rich way and were flexible about adapting them to the learners and the situation.

*“So, I find that it also allows you to put yourself more at the level of the intern or trainee you have in front of you. It also allows... this is a bit of a hypothesis, but I think it allows you to create a sort of reflection strategy in the person opposite. If we verbalise out loud the questions we ask ourselves or the direction we want the reflection to take, it’s then, I have the impression, something like an instruction manual that he’ll be able to re-use more than if we say to him: “well, you see in this case, we would have done this, this, this, you think like this, like that, you think like that.” (CT13: line 39)*

Their skills and attitudes stemmed from multiple experiences. They tended to replicate what they appreciated about their own role models, and they had attended faculty development training programs. They also enriched their teaching framework with other frameworks such as therapeutic education and motivational interviewing.

When teaching clinical reasoning, they demonstrated accurate analytical skills and tried to adapt to what the learner said. They worked together to co-construct a reasoning strategy without imposing their perspective in order to achieve a common understanding; they considered that solving the clinical problem was part of a learning process. They demonstrated mastery in both teaching vocabulary and concepts.

*“Then, he didn’t... he didn’t have a representation of the problem, and so, I think that he simplified the problem a little bit by saying what she had already had, etc., without already having a very clear diagnostic orientation. So, I would say, imagining the problem was not obvious for him. Generating hypotheses was challenging for him. Confronting them with the data collection was also difficult, and the management plan, we didn’t even get that far.” (CT14: line 56)*

Going to see the patient was described as useful to give observation tasks to the learner, demonstrate clinical skills, to do together, to debrief after and finally to reassure the patient.

#### Teaching styles in transition.

Finally, the remaining clinical teachers (n = 5) were described as in transition as their supervision style appearing could not fit the four styles and appeared to be in development. Most of them (n = 4) were trained and worked as family physicians.

Some had a rich framework acquired by training, private and professional experiences which led them to adopt a holistic approach of the learner. They emphasized flexibility and reflexivity in teaching skills but were unsure and self-critical about the way they put into action or use what they knew or had learned.

“Yes... well, I don’t know (Laughs.) Well, maybe that’s my experience too. I lived in countries where I arrived, I didn’t speak the language. So, there’s so much you can learn just by looking, it really put me back in that... That’s clear. And *if in Africa*... well, there’s an understanding that doesn’t come through questions. So, I don’t know. Maybe that’s it, but maybe that’s also my nature.” (CT5: line 56)

Others also had a rich teaching framework, mainly acquired through training and experience. It allowed them to analyse and diagnose the learner’s difficulties, but they struggled to implement effective teaching tools due to a lack of self-confidence.

The last participants had developed a very rich framework through self-training and experience and had perfectly mastered the clinical reasoning process allowing them to subtly analyse the reasoning difficulties of the learner. However, they had a rather rigid and binary way of supervising while being conscious of the need to be become more flexible.

*“ It is like a ball of wool and then we see a thread sticking out which says: “but look, he might be incompetent in his knowledge of the disease”. And then we start to pull and this time we’ll ask him questions on purpose, a little bit about basic knowledge, we say to him: “but go ahead, tell me again what you know about this or that?” and then we’ll quickly realise that in fact, he’s missing a lot of basics.... So you have to remain benevolent, you have to tell them “well listen, it’s not serious, if you’re here it’s because you have a... you’re someone who’s worthwhile and clever, but I think you lack basic knowledge and if you fill in your gaps, you’ll become really excellent and it’s a shame not to make the effort”. (CT10: line 63)*

### Learner dimension

Most participants reported that they would adapt their supervision according to the learner and whether they had worked with them before or not. They used such categorisation to judge the degree of autonomy and reliability of the learner. However, participants from the rich and flexible supervision style described that identifying where the learner was in their learning was important for tailoring their supervision accordingly and were more prone to explicitly inquire about the level of their learner.

*“So, he’s in his first year. I don’t know how many clinical cases he’s already seen… how many situations he’s handled on his own… so in the way he thinks, he still needs to use an analytical reasoning process with all the steps. He doesn’t yet have enough experience to use non-analytical reasoning, or even a mixed approach, because that only comes later with experience.” (CT14: line 20)*

For participants with other supervision styles, exposure to unexperienced learners was correlated with more red flags and more negative emotions but it did not prompt them to seek more information about the learner.

Clinical teachers reacted differently to the learner’s attitude when they challenged their suggestions (e.g., to order further investigations). Some clinical teachers having a simple and flexible style felt quickly destabilized or upset.

*“It’s the kind of comment that’s generally quite annoying — when the resident sees us as someone who worries too much… Because we ourselves are constantly, you know, wondering, ‘Am I overdoing it?” (CT6: line)*

Those with the simple and fixed style felt annoyed and were more prone to consider learners to be arrogant, judging, or uninterested.

*“When I see someone who’s nonchalant, and I’ve tried once or twice, but I can see he is completely uninterested, I lose the motivation I had to invest in that person. Basically, I just push them out of my field of view.” (CT 9: line 9)*

Again, such differences of perceptions did not seem to be linked to the amount of teaching experience.

### Context dimension

Most participants acknowledged that the place of work influenced the style and focus of the supervision. Unsurprisingly, teaching in the emergency room meant little time and high prioritization needs and participants working in in disciplines requiring rapid identification and management of emergent issues were more prone to use a simple and fixed teaching style.

*“At the emergency department, I find it difficult because we have so little time. The supervision is… it’s true that it’s less than when we’re on the wards, where we have more time to dedicate to them. And since what we’re focused on is diagnosing the illness… we don’t necessarily think to tell them how they could have presented things a bit better.” (CT11: line 22)*

Supervising after a patient admission on the ward (inpatient) or during scheduled patient encounters (outpatient) gave supervisors more time and allowed for a larger focus on differential diagnosis and management strategies and more inclusion of the mother’s opinion into the clinical reasoning process.

### Medicine and patient dimensions

The knowledge of medicine and patient was embedded within all dimensions and was difficult to analyse separately. However, it was especially the case when participants described how they taught the different steps of the clinical reasoning process, since they relied upon both on their own understanding of the patient’s profile and characteristics and their own clinical knowledge to guide their supervision content.

*“A 16-year-old girl comes in because she has abdominal pain — well, in my mind I’m thinking: ‘It could be a digestive issue, it could be gynecological, or it could be urinary,’ basically. And then I ask myself: ‘What do I need to know to confirm or rule out one of these diagnoses?’ That’s probably what I’ve already done by asking more specific questions, especially about the gynecological aspect. And then, by going back and forth between these elements, it helps us sort things out — like, ‘Is it acute gastroenteritis? Is it gynecological?’...” (CT1: line 46)*

## Discussion

The aim of this study was to explore how clinical teachers’ teaching scripts’ dimensions articulated with each other and were operationalized during supervision of clinical reasoning. We also evaluated whether the teaching scripts and their activation varied according to clinical teachers’ level of teaching experience and professional background.

We found the teaching scripts are operationalized into four main supervision styles that varied according to the richness of clinical teachers’ pedagogical dimension of teaching scripts as well as the flexibility of their teaching approach.

The richness of clinical teachers’ teaching scripts was influenced by several factors such as prior experiences as learners, prior teaching skills training, and reflective skills. The importance of prior clinical experiences has been highlighted in a study showing that clinical teachers’ beliefs, which influenced their teaching behavior, were largely informed by their experiences as a student [22]. The data collected in our study did not specify whether prior teaching training led to reflective skills or vice-versa. However, reflective practice is considered a key element of successful development programs because it results in more durable changes than skills practice alone through development of new insights and motivation for change [23] and contributes to personal growth [23,24].

Variations in supervision approaches have been reported in several studies and analyzed under several perspectives. Some described supervision styles according to the priority given to patient [10,12], perceived responsibility of care [11] or role identification [13]. More recently, Sheu pointed out differences between early, developing, and experienced supervisors in terms of trust and supervision [14]. These fit well with the Dreyfus’ model for expertise development from novice to expert [25]. The supervision styles we identified resembled several of these categorizations and mirrored the balance between security of care and teaching prevailing in different working contexts. However, our findings do not support a similar linear supervision development trajectory and ability as described by Sheu [14]. Some with very little teaching experience used a rich and flexible style while others with a long teaching experience still had a simple and fixed style. Other clinical teachers seemed to be in transition between different styles, independent of their level of teaching experience.

What parallels and differences can we draw between the development of illness script and teaching scripts? Several authors have outlined the similarities between clinicians’ use of clinical reasoning and a teacher’s use of educational reasoning [26], in terms of strategies used [27], and reliance of specialized knowledge organization [3,4,28,29]. Research has shown that as clinicians in training are progressively exposed to and are involved in management of more and more patients with various diseases and diseases presentations, their repertoire of illness scripts grows and enables them to manage various clinical situations at a relatively minimal cognitive expense [5]. Maturity develops when clinicians encapsulate lower-level, detailed concepts about causal and pathophysiological knowledge and their inter-relations under a small number of higher-level concepts [30]. Progress in clinicians’ knowledge organization and subsequent clinical reasoning can also be observed when clinicians transform the information collected from patients into more abstract concepts (semantic abstraction) [31].

Maturity in teaching scripts is reached when during the clinical supervision, clinical teachers are able to adequately identify clues to a student’s difficulty (common/habitual and transient or unusual), analyze them in light of the student’s level of competence and training needs, formulate a pedagogical diagnosis, and then choose or adapt a supervisory content and method that considers both these findings, the patient, and the context [32]. Regarding teaching scripts, we found that the richer the teaching script was, the more clinical teachers used semantic vocabulary and pedagogical concepts. This has been described as an indicator of teacher identity development [29]. They were also able to unfold both their clinical reasoning and teaching skills when describing and addressing the learner’s difficulty during stimulated recall interviews. From this perspective, the ability to unfold both the clinical and pedagogical skills (encapsulation process) seems to be a better indicator of the maturity of teaching scripts rather than an encapsulation process as described in the development of clinical scripts. Indeed, effective teaching of clinical reasoning requires being able to be explicit about one’s own clinical reasoning process, name the general clinical reasoning generic steps and explain to the patient the reasoning process in order to appropriately guide the learner acquire appropriate reasoning skills [29].

Finally, our findings also suggest that the clinical teachers’ working context – often closely linked to their professional background – seemed to influence the richness of their teaching scripts and shape their supervision styles. Supervision styles are said to reflect the way clinical teachers manage the tension between contextual triggers such as are learners’ competence, patient acuity, workload and teaching itself [12,33]. It is not surprising that participants working in emergency contexts or in disciplines requiring rapid identification and management of emergent issues were more prone to emphasize patient security and adopt a fixed supervision style. The fact that all clinical teachers categorized as “in transition” worked and supervised in a family medicine environment is of interest and suggests that the uncertainty expressed by some clinical teachers trained in family medicine may mirror the uncertainty prevailing in family medicine [34].

The context and the culture of the work environment together with the task complexity determine the amount of responsibility and autonomy given to the learner [35]. Clinical teachers are considered to be competent when they encourage learners’ development of competence and autonomy while keeping risks to an acceptable level [36].

### Consequences for faculty development

Although operationalizing of teaching scripts might differ according to the priorities of the working context, our findings have several implications for faculty development programs. As competency-based curriculum and assessment of entrustable professional activities are being increasingly implemented in the workplace training, analysis of supervision styles and factors influencing them such as clinical teachers’ tolerance to insecurity and their ability to trust learners according to patient acuity, task complexity, and the working environment should be addressed in faculty development programs.

Teaching styles and practices may also vary according to the way clinical teachers view their teaching role in relation to their identity as a teacher, clinician, mentor, and or researcher. Several authors have indeed shown that the way clinical teachers develop and negotiate their different identities (compartmentalized, hierarchical or merged) influence their motivation and teaching practices [37,38]. Clinical teachers’ identities evolve over time and are influenced by their faculty development training. It is possible that for some, their teaching identity was present from the beginning.

Clinical teachers are often unaware of the different dimensions of their teaching script and how they influence their supervision style. Because teaching scripts remain difficult to deconstruct as the different domains activated are closely intertwined, we would encourage clinical teachers to first self-reflect about their supervision styles, just as physicians are asked to analyze and reflect about their own leadership styles before developing new leadership skills [39]. Ideally, clinical teachers should be able to choose among different supervision styles according to learners’ needs and context priorities and be at ease with any style independently from their personal preferences. However, more specific actions can be privileged according to the supervision style identified among clinical teachers. For those who have a simple and flexible style, the aim will be to help them enrich their teaching frameworks through training in order to increase their self-confidence. For those having a simple and fixed style, the challenge will be to stimulate their self-awareness and reflective skills as they appear essential for adaptability and behavioral changes. For those with rich and fixed style, the focus could be put on how to put into practice what has been taught. Finally, for those with rich and flexible style, the aim will be to further progress, enrich and transmit. Knowledge of different styles may also help clinical teachers described as “in transition” to specify and affine their style according to the goal they want to achieve [10]. For clinical teachers using a fixed style or being in transition, OSTEs (practice of clinical teaching skills with a simulated resident), are a valuable teaching tool by allowing the creation of several authentic teaching scenarios, direct feedback, repeated practice, and the stimulation of reflection on performance [40].

### Strengths and limitations

The main strength was that we explored operationalizing of teaching scripts and analysis of supervision styles through a methodology which took into account both an observation of a simulated clinical supervision and clinical teachers’ reflections about such supervision. We also included participants from different professional backgrounds and levels of teaching experience.

However, there are several limitations to our study. The sample of participants remained small and inclusion of clinical teachers from other disciplines such as surgery and psychiatry may have allowed to potentially expand our understanding of teaching scripts and their influence on supervision styles. Because we based our analysis on the supervision of only one clinical presentation, we were not able to evaluate whether different clinical cases may have activated different teaching scripts and led to a variability of supervision styles among the same participants. Future studies should, therefore, include a larger sample and a different clinical context, which could deepen a discussion around each clinical teacher’s style in the context of patterns and responses to a range of situations rather than the one-off. Perhaps this could be explored as an opportunity or direction for future work. Finally, we did not evaluate standardized residents’ perceptions regarding the supervision they received. These elements are of importance since trainee’s characteristics are factors among others contributing to the variability of supervision styles and entrustment decisions [11].

## Conclusion

This study sheds light on the different dimensions of teaching scripts which are at play when clinical teachers supervise clinical reasoning in the workplace and the impact of prior learning experienced, reflective teaching skills training, and to some extent of the working context on clinical teachers’ supervision styles. Maturity in teaching scripts seems to be achieved when clinical teachers are able to deconstruct their own clinical and teaching approaches and adopt flexible learner-centred approaches considering both learners’ needs, the complexity of the task, and the working environment. In order to expand and refine clinical teachers’ supervision skills, faculty development programs should include development of reflective practice about supervision styles and factors influencing them in order to train effective clinical teachers in the workplace.

## Supporting information

S1 Appendix Appendix A Standardized resident instructions and prompts. (DOCX)

S2 Appendix Appendix B Interview guide for the Debriefing and Stimulated recall session. (DOCX)

S3 Appendix Appendix C Participants’ detailed socio-demographic characteristics. (DOCX)
