# Supplementary material for: Script or style? Analysis of the relationship between teaching scripts and supervision style
Source: PLoS One. 2026 Jan 8;21(1):e0338902. doi: 10.1371/journal.pone.0338902 (PMC12782432; doi:10.1371/journal.pone.0338902)
Supplement: S1 Appendix — (DOCX) [file pone.0338902.s001.docx]

S1 Appendix. Standardized resident instructions and prompts

| If the CT (clinical teacher) interrupts during the case presentation | The resident answers the question laconically and resumes his presentation (saying "where was I... ") |
| --- | --- |
| If the CT requests a gynecological history | Patient menstruates every month or so, has no contraception, does not currently have a boyfriend; menstruated last week (not specifically asked if sexually active at this time) |
| If the CT asks for the elements mentioned, awaiting for certain answers | Link between pain and...   - Link between pain and food: I don't know - Link between pain and defecation: not really - Relationship between pain and menstruation: not asked but unlikely - Relationship between pain and movement: don't know - Pain at night: not reported - Weight loss not asked - Anxiety or depressive symptoms not asked, no exploration of sleep disorders   Gynecology   - Menarche: not requested - Quantification of menstruation: not bad for the first 2 days then much less   Personal background   - Nothing special except for ankle sprains - Early scoliosis for which she had physio during puberty - No surgery (did not ask specifically if abdominal surgery)   What is she doing?   - She is in high school Clinical examination - Rectal examination not done (I don't see what it would have brought, she seemed embarrassed)   Respond “No” to anything else that is not in the text |
| What are you thinking about? | Difficult because there is nothing very clear in what she is telling me... I can't make sense of it |
| What could it have  *Answer in italics if the CT asks for an argument* | I'm thinking... constipation  *Pros: slower bowel movements*  *Cons: intensity of pain, from there to not going to school* |
| Anything else?  *Answer in italics if the CT asks for an argument* | I feel she is very stressed, a functional thing? it happens quite often at this age (like spastic colon)  *Arguments for: her parents are divorced, there may have been tension*  *Arguments against: 3-day duration only, it should be more chronic* |
| Are you thinking of a urine trail?  *Answer in italics if the CT asks for an argument* | Not really, she has no complaints, but maybe (not asked more if urinary complaints, see pain)  *Pros: urinary tract infection not very symptomatic*  *Arguments against: no alguria (hematuria and pollakiuria not asked)*  *And for a kidney stone the location is really not typical* |
| Could this be related to something gynecological? | Yes, it's not out of the question, but I don't really see it. The mother is worried and wonders if it is endometriosis, but I don't really believe it (don't know why the mother is worried about it, nor the daughter) If reiterating arguments about endometriosis: she didn't report pain that regularly... and I don't feel like it's at the same time as her period. |
| What else is gynecological?  *Answer in italics if CT asks for reasons* | If re-launch: I have nothing for a pregnancy, she has no boyfriend (not specifically asked if RS)  If second reminder: Ovarian problem, tubes, (does not mention PID)  Did not think about the risk of an ectopic pregnancy |
| Digestive infectious diseases?  *Answer in italics if CT asks for argument* | As it's been going on for a few days, without fever, I don't really see  *Argument for: but in fact, she has 37.6.*  *Argument against: But for appendicitis, I don't think so because it's not located on the right.* |
| What would you like to exclude? | I'm not sure  What if the CT says “fever”: an infection an inflammation  What if the CT says “appendicitis”: but I don't believe it is an appendicitis |
| If you had to sum up | 17-year-old girl, in good health, who has abdominal pains a bit vague since a few days and which prevented her from going to school with loss of appetite and a transit a bit slowed down. With the status of abdominal pains peri and sub umbilical and a sub febrile state. Nothing on the gynecological level but there is nothing very clear |
| She worries you | Kind of yes, but the mother seems quite worried. She tried to talk to me on the phone again but the call cut off. |
| If you had to prioritize | Difficult, I would put constipation, something psychological but at the same time it could be the beginning of a digestive infection or a gynecological problem |
| What would you do? | I would tend to let her in with a laxative like movicol or fig syrup |
| And if you had to do complementary exams | Really, I don't really see what should be done, for me it's functional  I hesitate between doing nothing or doing everything |
| If the CT insists that you do a workup | If you really want me to do a blood test (dubious-perplexed tone), then I would do a complete blood count with CRP, creat, liver tests, lipase a urine sediment; we can also do a PSA |
| To look for what? | So as not to miss something... if the CT says “like what”: an inflammation, an infection, something serious |
| And if everything is normal  If prompted | I don't see much, try an enema anyway?  *If asks for something else*: send her to the gynecologist?  She has already been to the gynecologist: no, I don't think so and it's an idea of endometriosis, I don't believe it |
| Something else | Why not, a scan? That would help rule out something serious |
| What do you take away from our discussion | Stay vague - examples to choose from  - That a check-up is still necessary  - That it could still be something serious  - There is still a lot to do  Etc. |
