# Supplementary material for: Script or style? Analysis of the relationship between teaching scripts and supervision style
Source: PLoS One. 2026 Jan 8;21(1):e0338902. doi: 10.1371/journal.pone.0338902 (PMC12782432; doi:10.1371/journal.pone.0338902)
Supplement: S2 Appendix — (DOCX) [file pone.0338902.s002.docx]

S2 Appendix. Interview guide for the Debriefing and Stimulated recall session

| **Debriefing interview guide** | **Stimulated recall interview guide and prompts** |
| --- | --- |
| - I'm just going to ask you a few questions about supervision before we watch the video together. - So, in general, how did the supervision go for you? (what went well, what went less well and why) - And according to you, what difficulties did the resident present? - What did you try to do about it? | - We will now move on to the second part of this interview/the stimulated recall interview. - The purpose of this interview is to get your feedback on important points from the supervision session: in your own words. - You will explain if you remember-what you were thinking at that moment/what reasoning you had/what your decision making/action process was. - Feel free to stop the video at other times as well, if you see other elements that you would like to comment on. We also reserve the right to stop the video, if we see other interesting moments that we would like your comment on. - 3 follow-up questions if necessary:  1. Description: "What's going on here?" (What do you mean by that?) 2. Explanation: "How did you come to think that? How do you explain this? What do you base this on? (Why do you think that?)" 3. Reaction: "What do you think? (if not happy: if you had to do it again, how would you do it? |
