# Supplementary material for: Script or style? Analysis of the relationship between teaching scripts and supervision style
Source: PLoS One. 2026 Jan 8;21(1):e0338902. doi: 10.1371/journal.pone.0338902 (PMC12782432; doi:10.1371/journal.pone.0338902)
Supplement: S3 Appendix — (DOCX) [file pone.0338902.s003.docx]

S3 Appendix. Participants’ detailed socio-demographic characteristics

| Teaching Pattern | ID | Gender | Age | Medical discipline | Level teaching experience | Pre-graduate training | Feedback skills training | Clinical reasoning teaching skills |
| --- | --- | --- | --- | --- | --- | --- | --- | --- |
| Simple and flexible | 1 | male | 36-40 | Internal medicine | 3-4 years | Geneva | Yes | Yes |
|  | 2 | female | 31-35 | Family medicine | 1-2 yrs | Geneva | Yes | No |
|  | 6 | male | 31-35 | Internal medicine | 3-4 years | Geneva | No | Yes |
|  | 11 | female | 26-30 | Internal medicine | 1-2 yrs | Geneva | No | No |
| Simple and fixed | 9 | male | 31-35 | Gyn-obstetrics | 1-2 yrs | Elsewhere | No | No |
|  | 16 | male | 31-35 | Internal medicine | 1-2 yrs | Geneva | Yes | Yes |
|  | 17 | female | 41-45 | Paediatrics | > 5 yrs | Elsewhere | Yes | Yes |
|  | 19 | male | 36-40 | Gyn-obstetrics | 3-4 years | Geneva and elsewhere | No | Yes |
|  | 20 | female | 56-60 | Gyn-obstetrics | > 5 yrs | Elsewhere | No | Yes |
| Rich and fixed | 8 | male | 36-40 | Internal medicine | > 5 yrs | Geneva | Yes | Yes |
|  | 12 | male | 41-45 | Internal medicine | > 5 yrs | Geneva | Yes | No |
| Rich and flexible | 3 | female | 36-40 | Family medicine | > 5 yrs | Geneva | Yes | Yes |
|  | 13 | female | 31-35 | Paediatrics | 1-2 yrs | Geneva | Yes | No |
|  | 14 | female | 41-45 | Family medicine | 3-4 years | Elsewhere | Yes | Yes |
|  | 18 | male | 31-35 | Internal medicine | 3-4 years | Geneva | Yes | Yes |
| In transition | 4 | female | 31-35 | Family medicine | 1-2 yrs | Elsewhere | Yes | Yes |
|  | 5 | female | 51-55 | Family medicine | 3-4 years | Swiss | Yes | Yes |
|  | 7 | male | 46-50 | Family medicine | > 5 yrs | Geneva | Yes | No |
|  | 15 | male | 46-50 | Family medicine | 3-4 years | Geneva | Yes | Yes |
|  | 10 | male | 47-50 | Paediatrics | > 5 yrs | Elsewhere | No | No |
